# Harmonizing Optimal Strategy for Treatment of coronary artery diseases – comparison of REDUCtion of prasugrEl dose or POLYmer TECHnology in ACS patients (HOST-REDUCE-POLYTECH-ACS RCT): study protocol for a randomized controlled trial

**DOI:** 10.1186/s13063-015-0925-5

**Published:** 2015-09-15

**Authors:** Joo Myung Lee, Ji-Hyun Jung, Kyung Woo Park, Eun-Seok Shin, Seok Kyu Oh, Jang-Whan Bae, Jay Young Rhew, Namho Lee, Dong-Bin Kim, Ung Kim, Jung-Kyu Han, Sang Eun Lee, Han-Mo Yang, Hyun-Jae Kang, Bon-Kwon Koo, Sanghyun Kim, Yun Kyeong Cho, Won-Yong Shin, Young-Hyo Lim, Seung-Woon Rha, Seok-Yeon Kim, Sung Yun Lee, Young-Dae Kim, In-Ho Chae, Kwang Soo Cha, Hyo-Soo Kim

**Affiliations:** Division of Cardiology, Department of Internal Medicine, Seoul National University Hospital, 101 Daehak-ro, Jongro-gu, Seoul, 110-744 Korea; Division of Cardiology, Ulsan University Hospital, University of Ulsan College of Medicine, Ulsan, Korea; Department of Cardiovascular Medicine, Regional Cardiocerebrovascular Center, Wonkwang University Hospital, Iksan, Korea; Chungbuk National University, Cheongju, Republic of Korea; Department of Internal Medicine and Cardiovascular Center, Presbyterian Medical Center, Jeonju, Republic of Korea; Cardiology Division, Kangnam Sacred Heart Hospital, Hallym University Medical Center, Seoul, Korea; Cardiovascular Center, St. Paul’s Hospital, The Catholic University of Korea, Seoul, Korea; Division of Cardiology, Yeungnam University Medical Center, Daegu, Korea; Cardiovascular Center, Seoul National University, Boramae Medical Center, Seoul, Korea; Division of Cardiology, Department of Internal Medicine, Dongsan Medical Center, Keimyung University College of Medicine, Daegu, Korea; Department of Cardiology, Soon Chun Hyang University Hospital Cheonan, Cheonan, Korea; Division of Cardiology, Department of Internal Medicine, College of Medicine, Hanyang University Medical Center, Seoul, Korea; Korea University Guro Hospital, Seoul, Republic of Korea; Department of Cardiology, Seoul Medical Center, Seoul, Korea; Inje University Ilsan Paik Hospital, Goyang, Republic of Korea; Department of Cardiology, Dong-A University Hospital, Busan, Korea; Seoul National University Bundang Hospital, Seongnam, Republic of Korea; Department of Cardiology, Pusan National University Hospital, Busan, South Korea

**Keywords:** Acute coronary syndrome, Prasugrel, Everolimus, Drug-eluting stent, Biodegradable implants

## Abstract

**Background:**

Antiplatelet treatment is an important component in optimizing the clinical outcomes after percutaneous coronary intervention (PCI) especially in patients with acute coronary syndrome (ACS). Prasugrel, which is a new P2Y12 inhibitor, has been confirmed as efficacious in a large trial in Western countries, and a similar trial is also to be launched in Asian countries. Although a 60-mg loading dose of prasugrel followed by 10 mg per day should be acceptable, there have been no data regarding the optimal dose in Asian patients. Furthermore, serum levels of prasugrel and the rates of platelet inhibition are known to be higher in Asians than Caucasians with the same dose of the drug.

Polymer, a key component of drug-eluting stents (DES), has been suggested as the cause of inflammation leading to late complications, and has driven many companies to develop biodegradable-polymer DES. Currently, there are limited data regarding the head-to-head comparison between BP-BES and the biostable polymer CoCr-EES or the newest platinum-chromium everolimus-eluting stent (PtCr-EES). Furthermore, the polymer issue may be more important in ACS where there is ruptured thrombotic plaque where polymer-induced inflammation may affect the local milieu of the stented artery.

Therefore, the present study dedicated only to ACS patients, will offer important information on the optimal prasugrel dose in the Asian population by comparing a 10-mg versus a 5-mg maintenance dose beyond 1 month after PCI, as well as giving important insight into the polymer issue by comparing BP-BES versus biostable-polymer PtCr-EES.

**Method/Design:**

Harmonizing Optimal Strategy for Treatment of coronary artery diseases – comparison of REDUCtion of prasugrEl dose or POLYmer TECHnology in ACS patients (HOST-REDUCE-POLYTECH-ACS) trial is a multicenter, randomized and open-label clinical study with a 2 × 2 factorial design, according to the type of stent (PtCr-EES versus BP-BES) and prasugrel maintenance dose (5 mg versus 10 mg), to demonstrate non-inferiority of PtCr-EES relative to BP-BES or the reduced prasugrel dose relative to conventional dose in an Asian all-comers PCI population presenting with ACS. Approximately 3400 patients will undergo prospective, random assignment separately to either stent or prasugrel arm (1:1 ratio, respectively). When the patients have contraindications to prasugrel, they are categorized into an antiplatelet observation group after stent-randomization. The primary endpoint is the patient-oriented composite outcome, which is a composite of all-cause mortality, any myocardial infarction (MI), any repeat revascularization in the stent arm at 12 months after index PCI. In the prasugrel arm, primary endpoint is any major adverse cardiovascular event, which is a composite of all-cause mortality, any MI, any stent thrombosis (Academic Research Consortium (ARC)-defined), any repeat revascularization, stroke, or bleeding (BARC class ≥ 2).

**Discussion:**

The HOST-REDUCE-POLYTECH-ACS RCT is the first study exploring the optimal maintenance dose of prasugrel beyond 1 month after PCI for ACS in Asian all-comers. In addition, this is the largest study dedicated only to ACS patients to evaluate the polymer issue in the situation of ACS by directly comparing biostable-polymer PtCr-EES versus BP-BES.

**Trial registration:**

ClinicalTrials.gov (ID: NCT02193971, 13 July 2014).

## Background

Acute coronary syndrome (ACS) is a leading cause of death and hospitalization in developed countries. In recent decades, the mortality and morbidities after ACS have been remarkably decreased with improvement in percutaneous coronary intervention (PCI) with a drug-eluting stent (DES) and treatment with antiplatelet agents [1, 2].

Prasugrel is a novel thienopyridine that irreversibly binds to the platelet P2Y12 receptor and inhibits adenosine diphosphate-induced platelet aggregation. Both agents are thienopyridine P2Y12 inhibitors, but prasugrel is more potent and has less variability in platelet inhibition than clopidogrel. In the TRITON-TIMI 38 trial enrolling ACS patients with planned PCI (mostly after diagnostic angiography), prasugrel significantly reduced ischemic events including stent thrombosis (ST) but increased major bleeding episodes, including fatal ones, compared with clopidogrel [3]. Currently, guidelines recommend prasugrel as an initial P2Y12 inhibitor over clopidogrel, unless the patients are older than 75 years of age, have low body weight (<60 kg), or previous history of transient ischemic attack or stroke [4]. Nonetheless, clinical efficacy and safety of prasugrel in Asians are still unclear. Studies have shown marked inter-ethnic difference in the pharmacokinetics and pharmacodynamics of prasugrel. Asians showed higher serum prasugrel levels with greater platelet inhibition than Caucasians at the same dose of prasugrel [5]. In terms of balance between bleeding and thrombosis, Asians have a lower risk of thrombotic event while a higher risk of bleeding in previous studies [6]. In addition, East Asians have not been properly enrolled in major clinical trials with prasugrel (7.8 % in TRIOLGY ACS, and 0.9 % in TRITON-TIMI 38) [3, 7]. In this regard, concerns need to be addressed in a dedicated trial of East Asians as to whether the currently recommended dose of prasugrel (60 mg loading/10 mg daily maintenance) is efficacious and also safe in East Asians.

In this study, we will compare 2 different maintenance doses of prasugrel beyond 1 month after PCI: conventional dose of 10 mg versus a reduced dose of 5 mg of prasugrel in Asian patients with ACS undergoing PCI. All of the enrolled patients will receive the currently recommended loading dose of prasugrel (60 mg), and maintain a 10-mg daily dose for the first month. After 1-month clinical follow-up, patients will receive maintenance dosage randomly assigned after PCI (10 mg versus 5 mg). Dual antiplatelet therapy is recommended for at least 1 year. Clinical adverse events, including bleeding, will be compared at 1-year clinical follow-up.

Another important issue is the efficacy and safety of DES in treating unstable coronary plaques of ACS patients. There had been a concern that DES may increase the risk of late or very late ST, and that DES use in treating unstable vulnerable plaques may lead to worse outcomes. However, many trials including the HORIZONS-AMI and the ACUITY trial have already confirmed efficacy and safety of DES even in ACS patients [8, 9]. Technological innovations have led to the development of second-generation DES with a thinner strut with biocompatible polymer, or third-generation DES with biodegradable-polymer. One of the representatives of second-generation DES with a biocompatible polymer is cobalt-chromium-based everolimus-eluting stent with fluorinated co-polymer coating (CoCr-EES). The CoCr-EES has shown to reduce repeat revascularization and ST in patients with ST elevation myocardial infarction (MI) compared to bare metal stents (BMS) [10]. Also, CoCr-EES (Xience V®, Promus®, Xience Prime®, Xience Xpedition®, Abbott Vascular, CA, Illinois, USA) has proved its excellent safety and efficacy in diverse populations with numerous registries, trials and network meta-analyses [11–15]. Otherwise, the biodegradable polymer-coated biolimus-eluting stent (BP-BES, Biomatrix®, Biomatrix Flex® and Nobori®, Biosensors, Newport Beach, CA, USA, and Terumo Corporation, Tokyo, Japan) adopted the polymer poly-D-L-lactic acid (PDLLA) which is fully metabolized within 6–9 months in the human body. It was developed with the aim of eliminating the inflammatory stimulus and to thereby reduce the risk of ST. BP-BES in reality has shown excellent safety in long-term outcomes. In the LEADERS trial, BP-BES showed a significantly lower rate of very late ST (>1 year) than the durable-polymer sirolimus-eluting Cypher stent [16]. Even in comparison with BMS, BP-BES showed better efficacy and a trend toward better safety in a dedicated trial for patients with ST-segment elevation MI (STEMI), COMFORTABLE AMI [17]. In network meta-analyses, however, BP-BES was consistently inferior to CoCr-EES in terms of safety [14, 15, 18]. The results of meta-analyses look somewhat different from the recent data of individual trials comparing CoCr-EES and BP-BES: for example, the NEXT trial or COMPARE II trial, which both showed comparable safety and efficacy between the two stents [19, 20]. This discrepancy might originate mostly from heterogeneity in the BP-BES group having 17 different BP-DES in the meta-analyses [21]. Currently, there are limited data regarding the head-to-head comparison between BP-BES and CoCr-EES or platinum-chromium-based everolimus-eluting stent (PtCr-EES). Therefore, we need more evidence to clarify whether polymer technology is important and influences clinical outcome. The polymer issue may be more important in ACS patients who have ruptured plaques and a coronary artery thrombosis where polymer may affect the microenvironment of the stented artery. In terms of metal, platinum-chromium alloy is the newest metal in the stent industry, is known as a very inert alloy, and may result in less vessel injury and inflammation after PCI.

Therefore, this randomized trial dedicated only to ACS patients, with a 2 × 2 factorial design will address: (1) whether the reduced (5 mg) maintenance dose of prasugrel is non-inferior to regular (10 mg) dose in maintenance period beyond 1 month after PCI for ACS, and (2) whether a newly-developed biostable polymer PtCr-EES (Promus Premier^TM^, Boston Scientific, Natick, MA, USA) is at least non-inferior to BP-BES (Biomatrix®, Biomatrix Flex®, or Nobori®, Biosensors, Newport Beach, CA, USA, and Terumo Corporation, Tokyo, Japan) in preventing target lesion failure in ACS patients.

## Methods

### Study objectives and hypotheses

The primary objectives of this study, with a 2 × 2 factorial design are: (1) to evaluate the safety and efficacy of 2 different maintenance doses of prasugrel beyond 1 month after PCI: the reduced maintenance dose of prasugrel (5 mg daily) versus conventional dose of prasugrel (10 mg) in ACS patients, and (2) to determine the safety and long-term efficacy of coronary stents with 2 different polymer technologies: biostable polymer PtCr-EES versus BP-BES. The working hypotheses of this study are: (1) that a reduced dose of prasugrel (5 mg daily) is non-inferior to a conventional dose (10 mg daily) in preventing major adverse cardiovascular events (MACE) at 12 months, and (2) that PtCr-EES is non-inferior to BP-BES in reducing patient-oriented composite outcome (POCO), defined as a composite of all-cause death, nonfatal MI, and any repeat revascularization at 12 months. MACE is defined as a composite of all-cause death, nonfatal MI, ST, repeat revascularization, cerebrovascular accident (CVA), and Bleeding Academic Research Consortium (BARC) class ≥ 2 bleeding [22].

### Study design

**Fig. 1:**
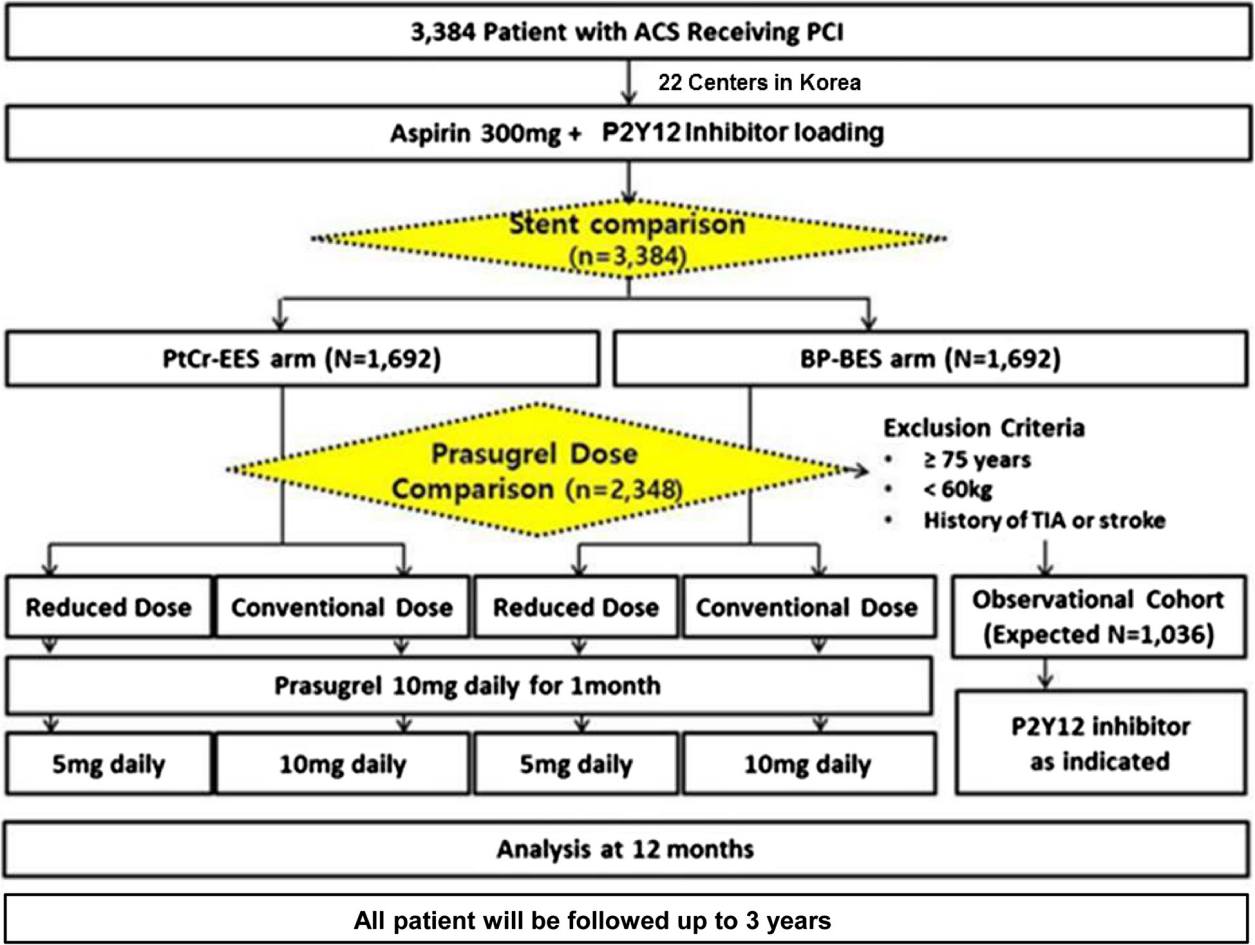
HOST-REDUCE-POLYTECH-ACS trial algorithm. *Abbreviations*: *ACS* acute coronary syndrome, *BP*-*BES* biodegradable polymer-coated biolimus-eluting stent, *PCI* percutaneous coronary intervention, *PtCr*-*EES* platinum-chromium-based everolimus-eluting stent, *TIA* transient ischemic attack

This is a prospective, open-label, multicenter, randomized controlled trial with a 2 × 2 factorial design. The flow chart of patients is shown in Fig. 1. The 27 cardiovascular centers in Korea will participate in this trial. Clinical follow-up will occur at 1, 12, 24, 36 months after index procedure. Investigators may conduct this follow-up as telephone interviews or office visits. There will be no mandatory angiographic follow-up unless clinically necessary. Prasugrel on-treatment platelet reactivity will be measured by using VerifyNow P2Y12 assay (Accumetrics, San Diego, CA, USA) in the antiplatelet arm at index procedure and at 1-month, and 12-month follow-up for the pre-specified subgroup analysis.

This trial is investigator-initiated, with grant support from Boston Scientific Korea, DIO co., and TERUMO Korea. Other than financial sponsorship, the companies have no role in protocol development or the implementation, management, data collection, and analysis of this study. The authors alone are responsible for design and execution of the trial, related statistical analyses, and all aspects of manuscript preparation, including drafting, editing, and final content. This study will be conducted according to principles outlined in the Declaration of Helsinki. All patients must provide written informed consent. This study protocol has been approved by the institutional review board of 22 participating centers in Korea. The study protocol was registered at www.clinicaltrials.gov (NCT02193971, https://clinicaltrials.gov/ct2/show/NCT02193971).

### Study population and entry criteria

ACS patients with at least 18 years of age who meet all of the inclusion criteria (Table 1) are eligible for this study. Patients must have a culprit lesion in a native coronary artery or a graft vessel with significant stenosis (>50 % by visual estimate) eligible for stent implantation as in the current recommendations of ACC/AHA/SCAI and ESC/EACTS guidelines [4, 23]. Patients with known hypersensitivity or contraindication to heparin, aspirin, clopidogrel, prasugrel, ticagrelor, biolimus, everolimus, or contrast media will be excluded. Additional exclusion criteria will be systemic (intravenous) biolimus, or everolimus use within 12 months, with active pathologic bleeding, with gastrointestinal or genitourinary bleeding within the prior 3 months, women of childbearing potential, history of bleeding diathesis, known coagulopathy (including heparin-induced thrombocytopenia), or refused blood transfusions, presence of non-cardiac co-morbid conditions with life expectancy less than 1 year or conditions that may result in protocol non-compliance. Inclusion and exclusion criteria will be graded to minimize exclusion of patients, thus reflecting a population at-large.

**Table 1:** Enrollment criteria

General inclusion criteria
1. Subject must be ≥ 18 years
2. Subject is able to verbally confirm understandings of risks, benefits and treatment alternatives of receiving percutaneous coronary intervention and he/she or his/her legally authorized representative provides written informed consent prior to any study related procedure
3. Subject must have a culprit lesion in a native coronary artery with significant stenosis (>50 % by visual estimate) eligible for stent implantation
4. Subject must have clinical diagnosis of acute coronary syndrome that includes unstable angina (crescendo, new-onset, resting) and myocardial infarction
Exclusion criteria
● The following patients will be enrolled in stent comparison, but excluded from antiplatelet prasugrel comparison. They will be classified as antiplatelet observational cohort:
1. Subjects ≥ 75 years
2. Body weight < 60 kg
3. History of TIA or stroke
1. The patient has a known hypersensitivity or contraindication to any of the following medications: heparin, aspirin, clopidogrel, prasugrel, ticagrelor, biolimus, everolimus, contrast media (patients with documented sensitivity to contrast media (e.g. rash) who can be effectively premedicated with steroids and diphenhydramine] may be enrolled. Those with true anaphylaxis to prior contrast media, however, should not be enrolled)
2. Patients with active pathologic bleeding
3. Gastrointestinal or genitourinary bleeding within the prior 3 months, or major surgery within 2 months
4. Systemic (intravenous) biolimus, or everolimus use within 12 months
5. Woman of childbearing potential, unless a recent pregnancy test is negative, who possibly plans to become pregnant any time after enrollment into this study
6. History of bleeding diathesis, known coagulopathy (including heparin-induced thrombocytopenia), or will refuse blood transfusions
7. Non-cardiac co-morbid conditions are present with life expectancy < 1 year or that may result in protocol non-compliance (per site investigator’s medical judgment)

*TIA* transient ischemic attack

If all eligible criteria are met and written informed consent is provided, patients will be randomized 1:1 to either (a) PtCr-EES or (b) BP-BES group, and 1:1 to either (a) reduced dose of prasugrel (5 mg daily) or (b) the conventional dose (10 mg daily) group. Patients who meet the exclusion criteria of prasugrel (age ≥ 75 years, body weight < 60 kg, or history of transient ischemic attack or stroke) will be enrolled in stent comparison, but excluded from prasugrel comparison. To generate comparable groups relative to known and unknown risk factors, randomization will be independently conducted online (T&W Software, Seoul, Korea) via web-based application. All randomization will be balanced, stratified by participating center and allocated treatment groups. According to the consideration of calculated sample size to meet non-inferiority in each comparison arms, a total of 3384 patients from 22 centers in Korea will be planned to be enrolled.

### Interventions with study device and drug

The treatment strategy will be determined by the study-certified interventional operator. It is recommended that each enrolling investigator review the most recently updated instructions for use (IFU) and assess the contraindications, warnings, and precaution sections for treating potential patients. Direct stenting or predilation, antithrombotic medications during the procedure, and use of glycoprotein IIb/IIIa inhibitors will be left to the operator’s discretion.

The PtCr-EES (Promus Premier^TM^) is available in diameters of 2.25, 2.5, 2.75, 3.0, 3.5 and 4.0 mm with lengths of 12, 16, 20, 24, 28, 32 and 38 mm (2.25 mm diameter not available for 38 mm lengths). The BP-BES (Biomatrix®, Biomatrix Flex®) is available in diameters of 2.25, 2.5, 2.75, 3.0, 3.5 and 4.0 mm with lengths of 8, 11, 14, 18, 24, 28, 33 and 36 mm (33 and 36 mm lengths not available for 2.25 and 4.0 mm of diameters). Another BP-BES (Nobori®) is available in diameters of 2.25, 2.5, 2.75, 3.0 and 3.5 mm with lengths of 8, 14, 18, 24 and 28 mm. The allocated stent from the randomization should be implanted in all lesions treated. However, other stents may be used in case of device failure or situations in which the operators decide considering the best interest of the patient. It is recommended to make two attempts to cross the lesion with the allocated stent before switching to another stent. Complete lesion coverage is recommended. In case of long lesions, stent overlap of 1 to 4 mm will be recommended per IFU. The use of intravascular ultrasound, fractional flow reserve or optical coherence tomography will be left to the clinician’s discretion.

All patients will receive 300 mg of aspirin and a loading dose of prasugrel intravenously before performing PCI. Patients excluded from antiplatelet comparison will receive other P2Y12 inhibitors including clopidogrel, and ticagrelor. Patients included in the antiplatelet comparison will maintain aspirin with 10 mg of prasugrel until 1-month follow-up. After a 1-month period, patients randomized to the conventional dose group will receive a 10-mg daily dose of prasugrel, and the reduced group will receive a 5-mg daily dose. Dual antiplatelet therapy will be recommended for at least 1 year. The antiplatelet agent, however, could be adjusted at the clinician’s discretion in patients with bleeding events.

After the 1-year follow-up visit, the patient could discontinue one of the dual antiplatelet agents in accordance with clinician’s discretion. Thus, we could evaluate the rebound phenomenon beyond 1 year after change of prasugrel to clopidogrel, as well as the clinical outcome in the chronic phase when the polymer has completely gone in the BP-BES.

### Outcome measurements and definitions

There are two primary endpoints in this study. In the stent comparison arm, the primary endpoint will be POCO, defined as a composite of all-cause death, nonfatal MI, and any repeat revascularization at 12 months. In the prasugrel dose comparison arm, the primary endpoint will be MACE, defined as a composite of all-cause death, nonfatal MI, ST, repeat revascularization, CVA, and BARC class ≥ 2 bleeding at 12 months. Revascularization is considered clinically driven with follow-up angiographic diameter stenosis ≥ 50 %, and at least one of the following: 1) history of recurrent angina pectoris, presumably related to the target vessel; 2) objective signs of ischemia at rest or during exercise testing (or equivalent), presumably related to the target vessel; 3) abnormal invasive functional diagnostic testing; or 4) target lesion or target vessel revascularization with ≥ 70 % diameter stenosis, even without aforementioned ischemic signs/symptoms [24, 25]. To reduce the chance of so-called “oculostenotic reflex,” whereby rates of repeated revascularization are disproportionately high, investigators will be recommended to perform a non-invasive stress test prior to the decision of the re-intervention of the target lesions as much as possible and they will adhere strictly to a clinically driven revascularization protocol. All clinical outcomes were defined according to the Academic Research Consortium (ARC) [25, 26]. All clinical events including revascularization will be independently adjudicated by an independent event adjudication committee, and only clinically driven revascularization will be coded as an event.

Secondary endpoints will be the individual components of the primary endpoints. In addition, POCO and MACE rate at 36 months, device-oriented composite endpoint (composite of cardiac death, target vessel MI, or target lesion revascularization), and acute procedure success (device, lesion and procedure) will be included as secondary endpoints. In prasugrel the comparison arm, adherence to the study dose of prasugrel, PRU value by VerifyNow P2Y12 at 1 month, 12 months, and the ratio of patients with the therapeutic range of P2Y12 reaction units (PRU, 86-238) will also be analyzed [27].

### Statistical considerations

#### Sample size calculations

Based on the event rates of a previous all-comer trial performed with the Korean population, we predicted POCO rates of PtCr-EES and BP-BES to be 6 % and 6 % at 12 months after index PCI, respectively [11, 13, 28]. Using a non-inferiority log-rank design with a non-inferiority margin of 2 %, sampling ratio of PtCr-EES:BP-BES at 1:1, allowing 5 % withdrawal rate in each group for the 12 months, a total of 3384 patients would result in a power of at least 81 % power with a 1-sided α of 2.5 %. The expected number of events in the stent comparison arm will be 362 as a total.

Also, to demonstrate the second hypothesis that the reduced dose of prasugrel is non-inferior to the conventional group regarding MACE rates at 12 months, we assumed the MACE rates of the reduced dose group and the conventional dose group to be 7 % and 8 %, respectively [29]. Using a non-inferiority design, a non-inferiority margin of 2.5 %, a sampling ratio of reduced dose group:conventional dose group at 1:1, allowing 5 % withdrawal rate in each group for 12 months, we need total 2348 patients in a statistical power of 75 % with a 1-sided α of 2.5 %. The expected number of events in the antiplatelet comparison arm will be a total of 342.

We expect 30 % of ACS patients’ criteria (age ≥ 75 years, body weight < 60 kg, or history of transient ischemic attack or stroke) would be excluded in the prasugrel comparison, which results in a sample size of 2369. If the number of exclusion criteria in the prasugrel comparison exceeds our expected number, patient recruitment will be done until the number of patients in the prasugrel arm is 2348.

#### Statistical analyses

All primary and secondary endpoints and serious adverse events will be analyzed after adjudication by a member of researchers or clinicians. All primary and secondary endpoints will be analyzed both on an intention-to-treat basis (all patients analyzed as part of their assigned treatment group), and per-protocol basis. For patients receiving multi-vessel PCI, the target lesion/vessel will be declared by the operator prior to the interventional procedure.

Primary endpoints will be analyzed firstly on an intention-to-treat basis (all patients analyzed as part of their assigned treatment group), and then, on a per-protocol basis at 12 months and 3 years after index procedure. The null hypothesis will be evaluated on a non-inferiority basis with Kaplan-Meier survival with the log rank test. Non-inferiority is defined by a 1-sided alpha of 2.5 % and a difference in net POCO rates of less than 2 % in the stent comparison arm and MACE rate of less than 2.5 % in the antiplatelet comparison arm. All primary and secondary endpoints will be analyzed on a per-patient basis. Events within the first month, the period receiving a 10-mg dose of prasugrel before taking the allocated prasugrel dose, will also be analyzed on an intention-to-treat basis. In addition, a landmark analysis will be performed at 1 month after index PCI, as a sensitivity analysis.

Device-oriented composite endpoint (target lesion failure: a composite of cardiac death, target-vessel MI, or target lesion revascularization) will be analyzed using the *χ*^2^-test and Kaplan-Meier survival with the log rank test. Other secondary endpoints including all-cause and cardiac death, target vessel/lesion revascularization, non-target vessel/lesion revascularization, any revascularization, target-vessel and all-cause (including non-target vessel) nonfatal MI, ST, stroke (ischemic or hemorrhagic), bleeding (BARC classification ≥ 2) will be analyzed using the *χ*^2^-test and Kaplan-Meier survival with the log rank test. Acute procedure success (device, lesion, and procedure) and adherence to study dose of drug (prasugrel) will be analyzed using the *χ*^2^-test. The baseline coronary angiographic characteristics will be analyzed on a per-lesion basis.

Patients who undergo VerifyNow P2Y12 assay will be also analyzed as a prespecified subgroup analysis. The PRU value will be analyzed using the *t* test n the conventional dose group versus reduced maintain dose group. The ratio of patients with a therapeutic PRU range (86–238 PRU) will be analyzed using the *χ*^2^-test.

#### Treatment of missing values

The primary analysis of the study endpoints will not be covariate-adjusted. No imputation methods will be used to infer missing values of baseline variables. For the study endpoints, patients lost to follow-up and subsequently lost to assessment of primary endpoint, will be considered to be censored in the estimation of Kaplan-Meier event rates. As a secondary analysis, we will also examine the patients who have been lost to follow-up. We will perform a comparison of baseline characteristics in patients with versus patients without 1-year follow-up. In addition, a sensitivity analysis will be performed to assess the impact of these patients on the study outcomes.

### Study organization and ethical consideration

The principal investigator, the study coordinator, and the Clinical Trials Center at Seoul National University Hospital (executive committee) are jointly responsible for all aspects of the study protocol and amendments. Site monitoring and data collection will be performed by a dedicated affiliated research coordinator. At appropriate intervals, designated trial monitors will review all investigational data for accuracy and completeness, ensuring protocol compliance. In addition to the Executive Committee, the Steering Committee, Data Safety and Monitoring Board, and the Clinical Event Adjudication Committee will be involved for the duration of the trial. This study was approved by the Institutional Review Board at Seoul National University Hospital (D-1404-142-576), Ulsan University Hospital, Wonkwang University Hospital, Chungbuk National University, Presbyterian Medical Center, and Kangnam Sacred Heart Hospital of Hallym University Medical Center, St. Paul’s Hospital, Yeungnam University Medical Center, Boramae Medical Center, Dongsan Medical Center, Soon Chun Hyang University Hospital Cheonan, Hanyang University Medical Center, Korea University Guro Hospital, Seoul Medical Center, Inje University Ilsan Paik Hospital, Dong-A University Hospital, Seoul National University Bundang Hospital, and Pusan National University Hospital. The protocol of the trial has been registered at http://register.clinicaltrials.gov (NCT02193971).

## Discussion

### Prasugrel dose after PCI

The antiplatelet agent is an important component of successful outcome after PCI. Prasugrel is a novel thienopyridine that irreversibly binds to the platelet P2Y12 receptor. It has faster acting, greater antiplatelet efficacy and more predictable effect than clopidogrel. The TRITON-TIMI 38 trial enrolled ACS patients with planned PCI and showed reduced recurrent cardiovascular events in the prasugrel group, compared with clopidogrel (from 11.2 % to 9.3 %, relative risk reduction 0.82, 9 % confidence interval (CI) 0.73–0.93, *p* = 0.002), mostly driven by a significantly lower risk of MI (from 9.2 % to 7.1 %, relative risk reduction 23.9 %, 95 % CI 12.7–33.7; *p* < 0.001). However, the rates of severe bleeding complications were higher in the prasugrel than the clopidogrel group (hazard ratio (HR) 1.32, 9 % CI 1.03–1.68, *p* = 0.03), driven mostly by an increased risk of spontaneous bleeding as well as fatal bleeding. Currently, ESC/EACTS guidelines recommend prasugrel as an initial P2Y12 inhibitor over clopidogrel, unless the patients are ≥ 75 years of age, with low body weight (<60 kg), or previous history of transient ischemic attack or stroke [4]. Nonetheless, the results from the RCT might not be representing daily real-world practice, especially within the specific populations: for example, the Asian population [30]. Actually, East Asians have not been properly enrolled in any major clinical trials with prasugrel [3, 7]. In addition, there has been much evidence demonstrating the significantly different pharmacokinetics and pharmacodynamics of prasugrel in the Asian population compared with the Western population [5, 6]. In that regard, there are concerns about universal application of the currently recommended dose of prasugrel (60 mg loading/10 mg daily maintenance) to the East Asian population.

In this study, we will compare the two different prasugrel maintenance regimens in Asian patients with ACS undergoing PCI. Patients will be randomized to 2 maintenance dosage regimens: 10 mg daily versus 5 mg daily. All of the enrolled patients will receive the currently recommended loading dose of prasugrel (60 mg), and maintain a 10-mg daily dose for 1 month. After 1-month clinical follow-up, patients will receive a maintenance dosage (10 mg versus 5 mg). Therefore, the HOST-REDUCE-POLYTECH-ACS trial will also provide valuable insight regarding the optimal maintenance dose of prasugrel to guarantee its potent antiplatelet effect as well as to minimize the potential hazard of bleeding complications in the Asian population. In the recent DAPT trial, change of prasugrel to clopidogrel at 30 months after PCI showed a mild increase of clinical events in the short- term period [31]. This cohort will be followed up for 3 years. Thus, we could also evaluate the rebound phenomenon beyond 1 year after change of prasugrel to clopidogrel in Asian people.

### Polymer issue in ACS patients: biostable-polymer PtCr-EES versus BP-BES

There has been concern that DES may increase the risk of ST, especially in the treatment of ACS patients. The main cause of this issue is the polymer, a key component of DES, because it may induce chronic inflammation in the stented artery, leading to thrombosis and restenosis especially in the ACS situation. With evolving technology, contemporary DES with thinner strut and biocompatible polymer consistently showed better efficacy and safety than previous first-generation DES [13–15]. Most of the evidence regarding second- generation DES with biostable polymer were generated from CoCr-EES. However, the newest metal, platinum-chromium alloy, is known as a very inert alloy, and may result in less vessel injury and inflammation after PCI, and emerges as a newer DES platform. Promus Premier^TM^ (Boston Scientific Korea, Seoul, Korea) is platinum-chromium-based everolimus-eluting stent, which adopted polyvinylidene fluoride-co-hexafluropropylene (PDVF-HFP) biocompatible polymer. The PDVF-HFP mimics the phospholipids on the outside of red blood cells and enables the polymer to induce minimal thrombus formation. These enhanced technologies might show at least non-inferior efficacy and safety, compared with third-generation BP-BES.

For the counterpart of the stent comparison arm, BP-BES adopted PDLLA which is fully metabolized within 6–9 months in the human body, for reducing inflammation and risk of ST. In the LEADERS trial, BP-BES was shown to be associated with a significant reduction in very late ST (5-year relative risk reduction 74 %, *p* = 0.003) and composite clinical outcomes compared to durable-polymer sirolimus-eluting stent [16]. In addition, BP-BES has shown better efficacy and a better safety trend than BMS in the COMFORTABLE AMI trial [32]. The pooled analysis of individual patient data from the ISAR-TEST 3, ISAR-TEST 4, and LEADERS trial also showed significantly reduced rates of ST with BP-BES, compared with the durable polymer sirolimus-eluting stent (HR 0.56, 95 % CI 0.35–0.95, *p* = 0.015), mainly driven by lower rates of very late ST (HR 0.22, 95 % CI 0.08–0.61, *p* = 0.004) [33].

Although biodegradable polymer technology has been highlighted as a new paradigm in developing DES, concerns are still present. Some studies revealed that BP-BES was also associated with inflammation and a cytotoxic effect as with other DES before polymer degradation [34, 35]. In addition, although recent head-to-head comparison between BP-BES with CoCr-EES showed comparable clinical outcomes up to 2 years of follow-up (NEXT trial) or up to 3 years of follow-up (COMPARE II trial), the COMPARE II trials showed a trend showing higher risk of ST in BP-BES than EES [18–20]. Moreover, currently available network meta-analysis consistently showed superior efficacy and safety of CoCr-EES, even to BP-BES. However, there are some controversies surrounding these network meta-analysis results. The included trials of the BP-BES group which showed significant diversity with the use of 17 different platforms of BP-BES: for example, Biomatrix®, Nobori®, Coracto, EucaTax, EXCEL, Yukon Choic PC, Jactax, Firehawk, SYNERGY, etc. [21]. Furthermore, meta-analyses have adopted clinical results at short-term follow-up as 1 year when polymer has not yet completely gone on BP-BES. Thus, controversies may originate from paucity of data regarding head-to-head comparison between BP-BES directly with second- generation DES for sufficient duration. In addition, the efficacy and safety of the BP-BES have not been compared directly with the second-generation biostable-polymer DES in the ACS situation where the polymer issue may be more critical. This cohort will be followed up for 3 years. Thus, we could evaluate the clinical outcome in the chronic phase when polymer has complete gone on BP-BES while remaining on PtCr-EES. Therefore, the trial will clarify the comparison of clinical outcomes between biostable polymer PtCr-EES and BP-BES and will provide valuable evidence regarding the optimal treatment strategy for the ACS patients.

After initiation of the ‘Harmonizing Optimal Strategy for Treatment of coronary artery diseases – comparison of REDUCtion of prasugrEl dose or POLYmer TECHnology in ACS patients’ (HOST-REDUCE-POLYTECH-ACS) trial, patient enrollment will be done within 2 years. This large and well-designed randomized controlled study will provide insights inton the two critical issues in PCI for ACS patients. First, it will answer the question regarding the optimal maintenance dose of prasugrel beyond 1 month after PCI by comparing 5 mg versus 10 mg of prasugrel in Asian ACS patients. Second, it will clarify the implication of the polymer issue in ACS patients by showing clinical outcomes of biostable polymer PtCr-EES versus BP-BES in real-world practice.

## Trial status

The HOST-REDUCE-POLYTECH-ACS randomized controlled clinical study with a 2 × 2 factorial design is currently ongoing. Patient recruitment was started in October, 2014 and follow-up is being conducted.

### Limitations

The possible expected limitations of the current trial should be acknowledged. Firstly, we set the statistical power of the prasugrel arm as 75 %. About 30 % of the stent comparison arm would not be enrolled in the prasugrel arm due to several factors (absolute or relative contraindication of prasugrel use, or other clinical circumstances including high risk of bleeding); therefore, sample size of prasugrel arm will be inevitably lower than stent comparison arm. With the limited sample size of the stent comparison arm, it was considered to be difficult to further enhance statistical power of the prasugrel arm in contemporary practice in Korea.

## Abbreviations

ACS: acute coronary syndrome
ARC: Academic Research Consortium
BARC: Bleeding Academic Research Consortium
BES: biolimus-eluting stent
BMS: bare metal stents
CAG: coronary angiography
CI: confidence interval
CoCr-EES: cobalt-chromium-based EES
CVA: cerebrovascular accident
BARC: Bleeding Academic Research Consortium
DES: drug-eluting stent
EES: everolimus-eluting stent
IFU: instructions for use
HR: hazard ratio
MACE: major adverse clinical events
MI: myocardial infarction
PCI: percutaneous coronary intervention
PDLLA: poly-D-L-lactic acid polymer
POCO: patient-oriented composite outcomes
PDVF-HFP: polyvinylidene fluoride-co-hexafluropropylene
PRU: P2Y12 Reaction Units
PRU P2Y12 Reaction Units PtCr-EES: platinum-chromium-based EES
ST: stent thrombosis
STEMI: ST-segment elevation myocardial infarction
